# Spermidine attenuates monocrotaline-induced pulmonary arterial hypertension with HADHA-associated improvements in fatty acid oxidation and mitochondrial redox homeostasis

**DOI:** 10.1016/j.redox.2026.104392

**Published:** 2026-09-17

**Authors:** Xinyu Bai, Keke Tian, Shiyuan Song, Xiuming Rao, Gang Xu, Erlong Zhang, Bao Liu, Yan Wang, Yuqi Gao

**Affiliations:** a Yu-Yue Pathology Scientific Research Center, Jinfeng Laboratory, Chongqing, 400039, China; b Institute of Medicine and Equipment for High Altitude Region, College of High Altitude Military Medicine, Army Medical University (Third Military Medical University), Chongqing, 400038, China; c Key Laboratory of Extreme Environmental Medicine, Ministry of Education of China, Army Medical University (Third Military Medical University), Chongqing, 400038, China

**Keywords:** Spermidine, Redox biology, Fatty acid β-oxidation, HADHA, Mitophagy, Pulmonary arterial hypertension

## Abstract

Metabolic reprogramming, impaired mitochondrial quality control, and mitochondrial redox imbalance are increasingly recognized as central drivers of pulmonary arterial hypertension (PAH). Spermidine (SPD) has been reported to exert cardiovascular protective effects; however, its effects on endothelial metabolism and redox homeostasis in PAH remain incompletely characterized. HADHA, the α-subunit of mitochondrial trifunctional protein, participates in long-chain fatty acid β-oxidation (FAO) and provides a potential link between fatty acid utilization, mitochondrial respiration, mitophagy-related quality control, and redox homeostasis. Here, using integrated metabolomics, lipidomics, and molecular biology approaches in monocrotaline (MCT)-induced PAH rats and palmitic acid-stimulated pulmonary microvascular endothelial cells (PMVECs), we examined the contribution of HADHA to the effects of SPD on endothelial FAO and mitochondrial redox homeostasis. SPD treatment markedly attenuated pulmonary hypertension and vascular remodeling, accompanied by reduced inflammatory activation and endothelial injury. Metabolomic and lipidomic profiling indicated that SPD partially corrected pathological lipid-metabolic remodeling, including phosphatidylethanolamine-associated lipid alterations, and improved FAO-related mitochondrial function under lipotoxic stress. SPD reduced lipid accumulation and lipotoxic mitochondrial injury, inhibited PLIN2-vimentin-associated endothelial-to-mesenchymal transition (EndMT) marker changes, and increased PINK1/Parkin-related mitophagy markers together with TOM20/LC3B/LAMP2 colocalization, consistent with improved mitophagy-related mitochondrial quality control. Molecular dynamics simulation, cellular thermal shift assay, and microscale thermophoresis supported SPD-HADHA binding and SPD-associated HADHA stabilization. Importantly, HADHA knockdown markedly attenuated several effects of SPD on FAO-related function, mitochondrial redox balance, and lipotoxic phenotypes in PMVECs. These findings suggest that HADHA may contribute to the effects of SPD on fatty acid oxidation, redox homeostasis, and endothelial protection in PAH.

## Highlights

• Spermidine attenuates experimental pulmonary arterial hypertension.
• Spermidine reduces mitochondrial oxidative stress and lipid peroxidation.
• Biophysical assays support direct spermidine-HADHA binding in vitro.
• HADHA knockdown attenuates several spermidine-associated protective effects.
• Spermidine improves mitophagy-related mitochondrial quality control.

## Graphical abstract

**Figure:**
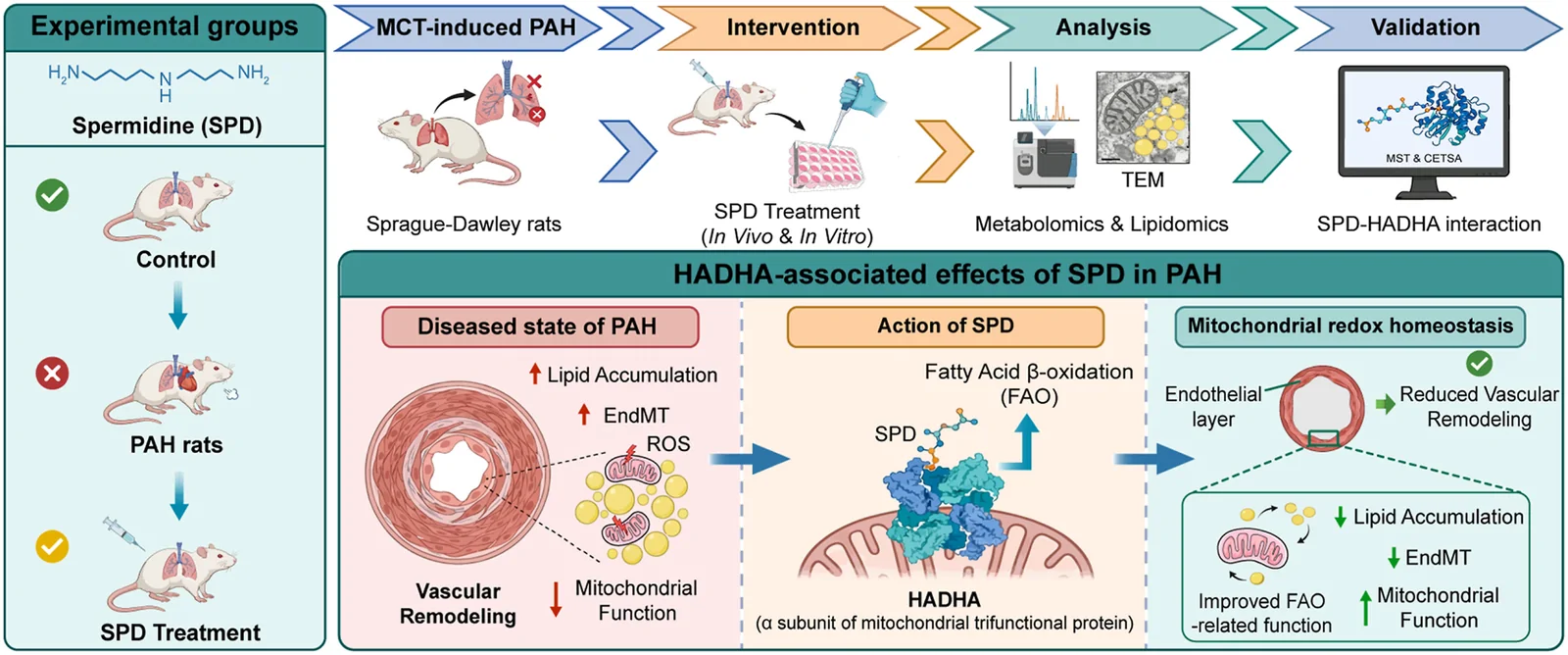

## 1 Introduction

Pulmonary arterial hypertension (PAH) is a chronic inflammatory vascular disorder characterized by sustained elevation of pulmonary arterial pressure, endothelial dysfunction, and vascular remodeling, ultimately culminating in right ventricular failure [1]. Endothelial injury is recognized as an early hallmark of PAH and a pivotal factor driving disease progression. This endothelial dysfunction manifests as reduced synthesis of vasodilators, such as nitric oxide, and abnormal release of vasoconstrictors like endothelin-1 (ET-1). These alterations result in persistent vasoconstriction, aberrant proliferation of vascular mural cells, and activation of inflammatory responses, collectively accelerating vascular remodeling and pulmonary hypertension [2]. Current therapeutic regimens, primarily comprising anti-proliferative agents and vasodilators (e.g., endothelin receptor antagonists and prostacyclin analogs), primarily alleviate symptoms rather than reversing established vascular remodeling. This limitation is evident from the persistently low seven-year survival rate, which remains below 50% [3,4]. Consequently, there is an urgent need for novel therapeutic strategies that address the underlying mechanisms of PAH and improve patient outcomes.

In monocrotaline (MCT)-induced experimental models, targeted endothelial injury initiates a cascade of interconnected events, including mitochondrial dysfunction, oxidative stress, and inflammation, which together promote endothelial apoptosis and compromise vascular integrity [5,6]. Beyond these immediate effects, injured endothelial cells undergo profound metabolic reprogramming. A shift toward glycolysis, increased dependence on glutamine metabolism, and excessive generation of reactive oxygen species (ROS) collectively exacerbate endothelial damage [1]. This sustained metabolic disorder and inflammatory stress ultimately drives endothelial cells to undergo a phenotypic transformation known as Endothelial-to-Mesenchymal Transition (EndMT). During this transition, cells downregulate endothelial-specific markers, such as CD31, VE-cadherin (CD144), while upregulating mesenchymal markers, including α-smooth muscle actin (α-SMA), and vimentin [7]. Importantly, EndMT is not merely a phenotypic alteration; these transformed cells actively contribute to neointimal formation and vascular thickening, providing a mechanistic link between endothelial injury and pathological vascular remodeling in PAH.

Spermidine (SPD) is a naturally occurring polyamine implicated in autophagy regulation, mitochondrial homeostasis, and cardiovascular protection [8]. Recent work suggests that SPD may attenuate MCT-induced PAH [9], but its effects on endothelial metabolism and redox homeostasis remain insufficiently characterized. Because PAH-associated endothelial injury is accompanied by lipid metabolic disturbance, mitochondrial dysfunction, and oxidative stress, identifying molecular nodes that connect fatty acid metabolism to mitochondrial redox homeostasis may help clarify how SPD protects the pulmonary endothelium.

HADHA is the α-subunit of mitochondrial trifunctional protein, which catalyzes key reactions in long-chain fatty acid β-oxidation [10]. Previous studies have shown that TFPα/HADHA is required for fatty acid β-oxidation and mitochondrial lipid remodeling, while endothelial fatty acid oxidation contributes to vascular protection through redox homeostasis [10,11]. In pulmonary hypertension, PPARγ-associated fatty acid oxidation has also been implicated in metabolic remodeling and right heart dysfunction [12]. Moreover, prior work in CD8^+^ T cells reported that spermidine can enhance mitochondrial trifunctional protein (MTP) activity and FAO-related metabolic function, providing a rationale, but not direct evidence in PMVECs, for examining the contribution of MTP/HADHA to the effects of SPD [13]. However, the role of HADHA in the effects of SPD on fatty acid oxidation and mitochondrial redox homeostasis in pulmonary endothelial cells remains unclear. In this study, using MCT-induced PAH rats and PA-stimulated PMVECs, we integrated metabolomics, lipidomics, redox imaging, mitochondrial functional assays, and target-validation approaches to examine the relevance of HADHA to the effects of SPD under PAH-related endothelial lipotoxic stress.

## 2 Methods

### 2.1 Animals and in vivo phenotyping

A total of twenty-four male Sprague-Dawley rats (6–8 weeks old, 200 ± 20 g) were randomly assigned to three experimental groups (n = 8 per group): Control, MCT, and MCT + SPD. Rats in the Control group received a single subcutaneous injection of an equal volume of normal saline. Rats in the MCT group received a single subcutaneous injection of monocrotaline (MCT, 60 mg/kg) to induce PAH. Rats in the MCT + SPD group received the same MCT injection followed by daily intraperitoneal administration of SPD (5 mg/kg/day) starting on the day of MCT injection. The Control and MCT groups received equal volumes of vehicle. All interventions lasted for 28 days. An additional Control and Control + SPD (5 mg/kg/day) cohort was evaluated as a supplementary in vivo baseline-control experiment, and the corresponding data are presented in Fig. S1.

At the end of the 28-day experimental period, rats were anesthetized for hemodynamic measurement. Right ventricular systolic pressure (RVSP) and mean pulmonary arterial pressure (mPAP) were recorded using a PowerLab 4/35 data acquisition system (PL3504, ADInstruments, Castle Hill, NSW, Australia) connected to a catheter inserted into the right ventricle via the right jugular vein. After hemodynamic measurement, blood was collected from the abdominal aorta, and the heart was excised. The right ventricle (RV) was separated from the left ventricle plus septum (LV + S), and the Fulton index was calculated as RV/(LV + S).

Lung and heart tissues were fixed in 4% paraformaldehyde, embedded in paraffin, and sectioned at 5 μm. Hematoxylin and eosin (H&E) staining was used to examine pulmonary arteriole and right ventricular morphology. Masson staining was performed to assess pulmonary arteriolar fibrosis. The medial wall thickness of pulmonary arterioles was calculated as medial wall area/total vessel area × 100%. All animal procedures were approved by the Institutional Animal Care and Use Committee of Yu-Yue Pathology Scientific Research Center (Approval No. CARE-YYLAB-D-2026-001) and were conducted in accordance with the National Institutes of Health Guide for the Care and Use of Laboratory Animals.

### 2.2 Cell culture, treatments, and RNA interference

Primary rat pulmonary microvascular endothelial cells (PMVECs) were cultured in complete endothelial cell medium (Zhong Qiao Xin Zhou Biotechnology, Shanghai, China) at 37°C in a humidified atmosphere containing 5% CO_2_. Cells were seeded at densities appropriate for downstream assays. For immunofluorescence staining, cells were plated in 24-well plates at 1 × 10^4^ cells/mL. For Western blot analysis, cells were plated in 10-cm dishes at 2 × 10^5^ cells/mL. After cell attachment, a lipotoxicity model was established by treatment with 100 μM palmitic acid (PA; Sigma-Aldrich, St. Louis, MO, USA) for 12 h. Cells were then treated with 5 μM or 10 μM SPD (Shanghai Yuanye Bio-Technology, Shanghai, China) for an additional 12 h.

For gene knockdown experiments, siRNAs were designed and synthesized by GenePharma (Suzhou, China). The siRNA sequences were as follows: PLIN2 sense, 5′-GAAGCAAAGAUACUAUGAATT-3′ and antisense, 5′-UUCAUAGUAUCUUUGCUUCTT-3′; HADHA sense, 5′-GCCCAAUUCAAAGGUAAAUTT-3′ and antisense, 5′-AUUUACCUUUGAAUUGGGCTT-3′. A scrambled negative-control siRNA was used as the control. Transfection was performed using Lipofectamine 3000 reagent according to the manufacturer's instructions.

### 2.3 Biochemical assays and colorimetric FAO measurement

Serum concentrations of IL-6, von Willebrand factor (vWF; Elabscience Biotechnology, Wuhan, China), and ET-1 (ImmunoWay Biotechnology, Jiangsu, China) were measured using ELISA kits according to the manufacturers’ protocols. Free fatty acid levels in serum and lung tissue homogenates were determined using a colorimetric assay kit (Nanjing Jiancheng Bioengineering Institute, Nanjing, China). Fatty acid synthase concentrations were quantified using ELISA kits (Chongqing Mengbio Biotechnology, Chongqing, China). The fatty acid β-oxidation (FAO) capacity of mitochondria in PMVECs was assessed using a colorimetric FAO assay kit (Elabscience Biotechnology, Wuhan, China). Optical density values were measured using a Multiskan GO 1510 full-wavelength microplate reader (Thermo Fisher Scientific, Waltham, MA, USA).

### 2.4 Mitochondrial respiration and seahorse-based FAO assessment

For high-resolution mitochondrial respiration analysis, PMVECs were stimulated with 100 μM PA followed by treatment with 5 μM or 10 μM SPD. After 24 h, cells were collected and counted. A total of 1 × 10^6^ cells were transferred into each chamber of an Oxygraph-2K high-resolution respirometer (OROBOROS Instruments, Innsbruck, Austria) at 37°C. Basal respiration was first recorded. Oligomycin was then titrated to determine respiration associated with proton leak. Maximal respiratory capacity was assessed by titrating carbonyl cyanide 3-chlorophenylhydrazone (CCCP), and rotenone was added to determine residual non-mitochondrial oxygen consumption.

Endogenous FAO in PMVECs was assessed using an XFe96 Seahorse Analyzer (102340-100, Agilent). PMVECs were seeded in Seahorse XFe96 cell culture microplates at a density of 3.0 × 10^4^ cells per well and subjected to the indicated treatments. Before OCR measurement, cells were incubated for 1 h under serum-free conditions in assay medium supplemented with 17.5 mM glucose, 2.5 mM glutamine, and 0.5 mM pyruvate. FAO-associated mitochondrial respiration was then measured using the Seahorse XF Long Chain Fatty Acid Oxidation Stress Test Kit (103672-100, Agilent) according to the manufacturer's instructions. Oligomycin, FCCP, and rotenone/antimycin A were sequentially injected during the assay, with FCCP used at a final concentration of 1 μM. The acute respiratory response to etomoxir treatment and the maximal respiratory capacity in the presence or absence of FAO inhibition were recorded and analyzed using Seahorse Wave software.

### 2.5 Histological, immunofluorescence, lipid-droplet, and redox staining

For lipid-droplet staining in lung tissue, fresh samples were embedded in optimal cutting temperature compound and snap-frozen. Cryosections were stained with BODIPY 493/503 to visualize lipid droplets. For immunofluorescence analysis, paraffin sections of lung tissue and PMVEC cell slides were fixed with 4% paraformaldehyde, permeabilized with 0.1% Triton X-100 in PBS, blocked with 5% goat serum, and incubated with primary antibodies at 4°C overnight. Samples were then incubated with the corresponding fluorescent secondary antibodies. Antibody information is provided in Supplementary Table S1.

Mitochondrial ROS production was assessed using MitoSOX staining. After the indicated treatments, PMVECs were incubated with MitoSOX reagent under the manufacturer-recommended staining conditions at 37°C in the dark. Cells were then washed with PBS and imaged using a confocal microscope. Lipid peroxidation was evaluated using C11-BODIPY 581/591 staining. After treatment, PMVECs were incubated with C11-BODIPY 581/591 under the manufacturer-recommended staining conditions at 37°C in the dark, washed with PBS, and imaged by confocal microscopy.

### 2.6 Transmission electron microscopy

PMVECs treated with PA and/or SPD were fixed with 2.5% glutaraldehyde in 0.1 M phosphate buffer (pH 7.4) at 4°C for 2 h. After post-fixation with 1% osmium tetroxide, samples were dehydrated through a graded ethanol series and embedded in epoxy resin. Ultrathin sections of 70 nm were cut, stained with uranyl acetate and lead citrate, and examined using a Tecnai G2 20 TWIN transmission electron microscope (FEI Company, Hillsboro, OR, USA) to evaluate mitochondrial morphology and lipid accumulation.

### 2.7 Western blot analysis

Total protein was extracted from homogenized lung tissue and PMVECs using ice-cold RIPA lysis buffer. Protein concentration was determined using a BCA protein assay kit. Equal amounts of protein (30 μg) were separated by 7.5-15% SDS-PAGE and transferred onto 0.22 or 0.45 μm nitrocellulose membranes (Merck Millipore, Burlington, MA, USA). Membranes were blocked with 5% skimmed milk for 1 h, incubated with appropriately diluted primary antibodies at 4°C overnight, and then incubated with corresponding secondary antibodies at room temperature for 1 h (for antibody details, see Supplementary Table S1). Protein bands were visualized using a hypersensitive ECL substrate and imaged with a chemiluminescence imaging system (ChemiScope S, Shanghai Clinx Scientific Instrument, Shanghai, China). Band intensities were quantified using ImageJ software (version 1.54, National Institutes of Health, USA) and normalized to β-actin.

### 2.8 GST pull-down assay

Glutathione S-transferase (GST) recombinant proteins were expressed in Transetta (DE3) *E. coli* cells transformed with the pGEX-6p-1 plasmid encoding GST-PLIN2_189–411_. Protein expression was induced with 0.5 mM isopropyl β-d-1-thiogalactopyranoside at 37°C for 3 h. Bacterial lysates were purified using GST-tag purification resin (Beyotime Biotechnology, Beijing, China), and GST-PLIN2_189–411_ expression was verified by SDS-PAGE and Coomassie blue staining. For pull-down assays, equal amounts of GST or GST-PLIN2_189–411_ were incubated with His-tagged vimentin (TargetMol). After washing, bound proteins were eluted and analyzed by western blotting using anti-His and anti-GST antibodies.

### 2.9 CETSA and MST analyses

To assess whether SPD alters the thermal stability of HADHA in cell lysates, a cellular thermal shift assay (CETSA) was performed. PMVECs were collected and lysed in NP-40 lysis buffer (Beyotime Biotechnology, Shanghai, China) at 4°C for 1 h. Lysates were centrifuged at 20,000 g for 15 min at 4°C, and the supernatants were collected. One aliquot was supplemented with SPD at a final concentration of 10 μM, whereas the other received an equal volume of PBS. After incubation at room temperature for 1 h with gentle shaking, samples were divided and heated at 43, 46, 49, 52, 55, 58, 61, 64, 67, or 70°C for 3 min. Samples were then centrifuged at 20,000 g for 20 min at 4°C, and the supernatants were collected for Western blot analysis.

Microscale thermophoresis (MST) was performed to determine the binding affinity between SPD and recombinant human HADHA. Recombinant human HADHA protein was purchased from abinScience (Cat. No. HW371012) and fluorescently labeled using a RED-NHS protein labeling kit (NanoTemper, MO-L011) according to the manufacturer's instructions. Briefly, the protein buffer was exchanged into labeling buffer, and the HADHA concentration was adjusted to 5 μM before labeling. HADHA was incubated with RED-NHS dye at room temperature in the dark for 30 min, and excess dye was removed by size-exclusion purification. The labeled HADHA protein was then mixed with serially diluted SPD in assay buffer containing 0.05% Tween-20 and incubated at room temperature for 20 min. Samples were loaded into standard treated capillaries (NanoTemper, MO-K022), and MST signals were recorded using a Monolith NT.115 instrument. Binding curves and Kd values were analyzed using NanoTemper analysis software.

### 2.10 Targeted metabolomics and lipidomics profiling

Targeted metabolomics was performed using the H800 targeted metabolomics platform by Shanghai Applied Protein Technology Co., Ltd. (Shanghai, China). The H800 platform covers 765 measurable metabolites. Lung tissue samples were extracted using cold methanol/acetonitrile/water extraction solvent (2:2:1, v/v/v) containing stable-isotope internal standards. Briefly, tissue samples were homogenized, sonicated at 4°C, and centrifuged at 14,000 g for 20 min at 4°C. The supernatants were dried in a vacuum centrifuge and reconstituted in acetonitrile/water (1:1, v/v). After centrifugation at 14,000 g for 15 min at 4°C, the supernatants were subjected to LC-MS/MS analysis.

H800 metabolomic analysis was performed using an Agilent 1290 Infinity LC UHPLC system coupled to a 6500+ QTRAP mass spectrometer (AB SCIEX). Metabolites were separated using HILIC and C18 columns. For HILIC separation, a Waters UPLC BEH Amide column was used, and for reversed-phase separation, a Waters UPLC BEH C18 column was used. Mass spectrometric detection was performed in positive and negative electrospray ionization modes with multiple reaction monitoring (MRM) acquisition. Raw MRM data were processed using MultiQuant or Analyst software. Metabolite quantification was based on peak extraction, peak-area/internal-standard ratios, and calibration curves. Metabolite annotation was based on retention time, MRM transitions, standard compounds, and database information, including KEGG ID, HMDB ID, superclass, class, subclass, and direct-parent annotations. After peak extraction, metabolite annotation, and quality-control assessment, the final H800 matrix used for downstream statistical analysis contained 500 measured metabolites.

Targeted lipidomics was performed using the HL2400 targeted lipidomics platform by Shanghai Applied Protein Technology Co., Ltd. Lipids were extracted from PMVEC samples using the methyl tert-butyl ether method with internal standards. The organic phase was collected, dried, and reconstituted before LC-MS/MS analysis. Lipidomic analysis was performed using a UHPLC system coupled to a 6500+ QTRAP mass spectrometer (AB SCIEX). Lipids were separated using C18 and Amino columns and detected in positive and negative electrospray ionization modes with MRM acquisition. Raw MRM data were processed using Sciex OS software. Lipid quantification was based on chromatographic peak integration and internal standards. Lipid annotation was based on retention time, MRM transitions, the targeted lipid library, lipid class annotation, and internal standards. The HL2400 targeted lipid library used for annotation contained 2464 candidate lipid entries across six major lipid categories and 25 lipid classes. After peak extraction, lipid annotation, and quality-control assessment, the final lipidomics matrix used for downstream statistical analysis contained 2171 lipid species.

For both targeted metabolomics and lipidomics datasets, pooled quality-control samples were inserted into the analytical sequence to evaluate system stability and data reproducibility. Quality-control assessment included total ion chromatogram overlap, QC sample clustering in PCA, Pearson correlation analysis, Hotelling's T^2^ analysis, multivariate control charts, and QC RSD/CV assessment. PCA was used as an unsupervised overview of global sample distribution. OPLS-DA was used to calculate VIP values, and differential metabolites or lipid species were selected using VIP > 1, |log_2_FC| > 1, and *P* < 0.05. The analytical workflows for the H800 targeted metabolomics and HL2400 targeted lipidomics datasets are summarized in Supplementary Table S2.

### 2.11 Molecular docking and molecular dynamics simulations

The three-dimensional structure of SPD was obtained from the PubChem database and optimized using AutoDockTools, including energy minimization, charge assignment, and format conversion. Molecular docking was performed using AutoDock Vina to evaluate the potential binding mode of SPD with HADHA (PDB ID: 5ZRV). The docking grid box was centered on the predicted binding region of HADHA. To further evaluate the stability of the SPD-HADHA complex, a 100-ns molecular dynamics simulation was conducted using the Desmond module of the Schrödinger software package. Trajectories were analyzed for binding free energy, root mean square deviation, root mean square fluctuation, hydrogen bond stability, and principal component analysis.

### 2.12 Statistical analysis

Data are presented as mean ± SD. All statistical analyses were conducted using GraphPad Prism (version 10.4.1). The number of independent biological replicates (n) for each experiment is detailed in the respective figure legends. Normality of data distribution was assessed using the Shapiro–Wilk test prior to inter-group comparisons. For comparisons across multiple groups with normal distribution, one-way analysis of variance (ANOVA) was employed. If the assumption of homogeneity of variance was satisfied, Tukey's post hoc test was utilized; otherwise, Dunnett's T3 test was used. A *P*-value of less than 0.05 was considered statistically significant.

## 3 Results

### 3.1 SPD ameliorates hemodynamic disorders and vascular remodeling in MCT-induced PAH

To investigate the therapeutic potential of SPD in PAH, we established the MCT-induced rat model. SPD treatment markedly ameliorated pulmonary arterial pressure in the PAH rats (Fig. 1A). Specifically, SPD significantly reduced RVSP, mPAP, and the Fulton index (Fig. 1B). These changes indicate attenuation of MCT-induced pulmonary hypertension and right ventricular remodeling. ELISA results showed that SPD significantly reduced MCT-induced IL-6 release and the levels of endothelial injury-related factors, including vWF and ET-1 (Fig. 1C). These observations suggest that the protective effect of SPD involves reducing systemic inflammation and endothelial damage. Histopathological analyses supported these findings. H&E and Masson staining revealed that SPD treatment reduced right ventricular hypertrophy, pulmonary arteriolar wall thickening, and attenuated collagen fiber deposition in PAH rats (Fig. 1D and E), in line with the observed improvement in hemodynamic parameters.

**Fig. 1:**
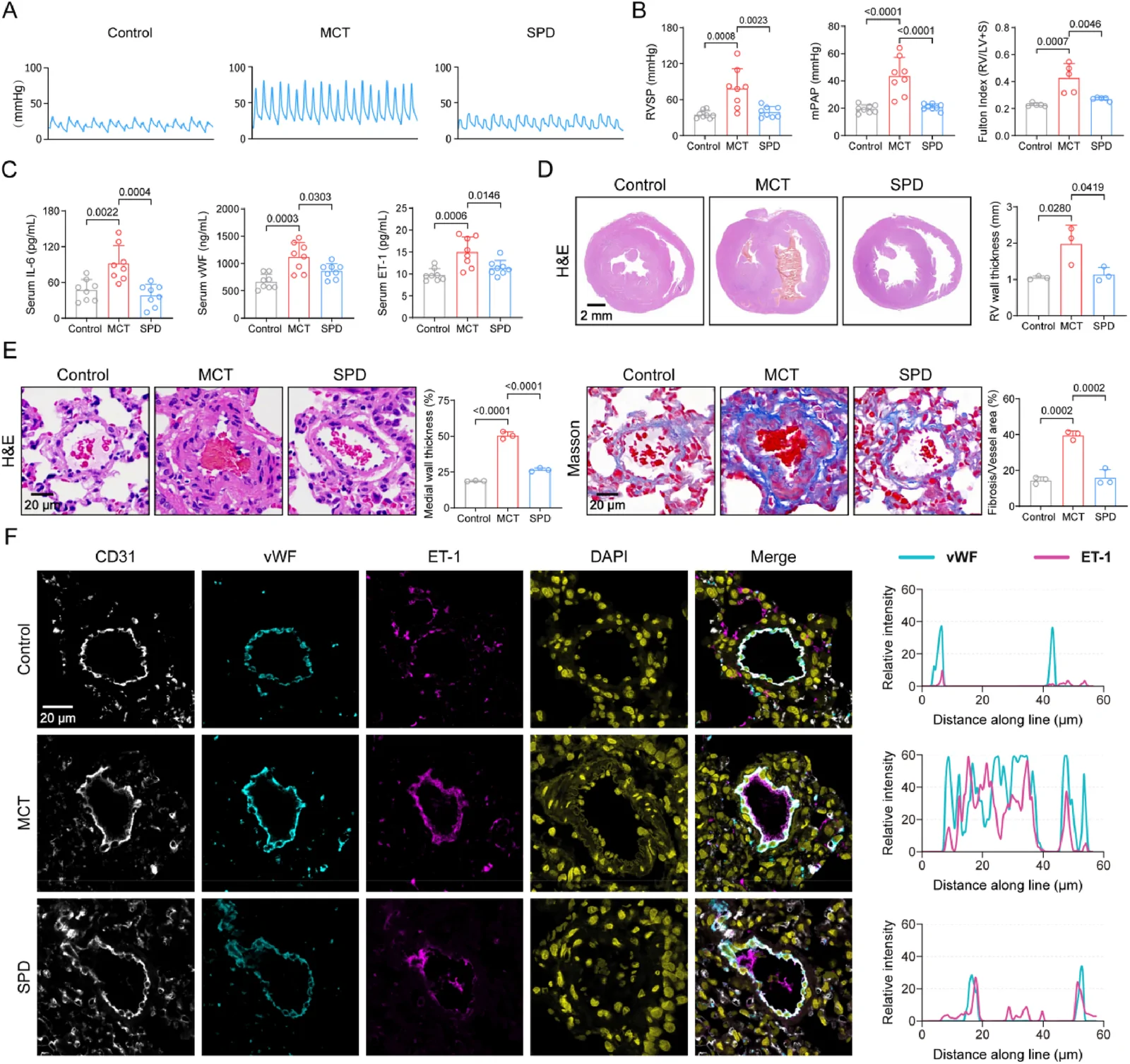
SPD attenuates MCT-induced PAH and endothelial injury. (A) Representative pulmonary arterial pressure recordings. (B) Assessment of RVSP, mPAP, and Fulton's index (Fulton's index n = 5, RVSP and mPAP n = 8). (C) Serum IL-6, vWF, and ET-1 levels (n = 8). (D) Representative H&E staining of heart sections and quantification of right ventricular remodeling (n = 3). (E) Representative H&E and Masson staining of lung sections and quantification of pulmonary arteriolar remodeling/fibrosis (n = 3). (F) Immunofluorescence staining of CD31, vWF, and ET-1 in pulmonary microvascular regions (n = 3). Data are presented as mean ± SD. For morphometric quantification, multiple vessels, microscopic fields, or tissue sections were measured from each animal. Measurements from the same animal were averaged to generate one animal-level biological replicate, and each plotted point represents one animal.

Finally, we focused on the endothelial cells within the pulmonary microarteries. Immunofluorescence staining (Fig. 1F) showed that the expression of endothelial injury markers (vWF, and ET-1) was significantly upregulated in the MCT group. SPD treatment effectively suppressed the abnormal expression of vWF and ET-1. Together, these systemic, histological, and endothelial-associated readouts indicate that SPD attenuates MCT-induced endothelial injury and pulmonary vascular remodeling. In an additional supplementary in vivo baseline-control experiment, SPD administration alone did not significantly alter the baseline physiological and hemodynamic parameters examined in healthy rats (Fig. S1), supporting that the effects observed in the MCT + SPD group reflect modulation of disease-associated pathology rather than nonspecific baseline disturbance.

### 3.2 SPD attenuates lipid metabolic remodeling and fatty acid dysregulation in MCT-induced PAH

To assess the metabolic alterations associated with SPD treatment in MCT-induced PAH rats, we performed high-throughput targeted metabolomics using the H800 platform. After peak extraction, metabolite annotation, and quality-control assessment, 500 measured metabolites were retained in the final H800 targeted metabolomics matrix for downstream analysis. Principal component analysis (PCA) showed partial separation with some overlap among the Control, MCT, and SPD groups, indicating a shift in the overall targeted metabolomic profile (Fig. 2A). We next performed pairwise differential metabolite analysis using VIP values derived from OPLS-DA models and the predefined criteria of VIP > 1, |log_2_FC| > 1, and *P* < 0.05. Volcano plots showed the differential metabolite profiles for the MCT versus Control comparison and the SPD versus MCT comparison (Fig. 2B). Based on the overlap between MCT-associated differential metabolites and SPD-regulated metabolites, 31 SPD-modulated MCT-associated differential metabolites were identified (Fig. 2C).

**Fig. 2:**
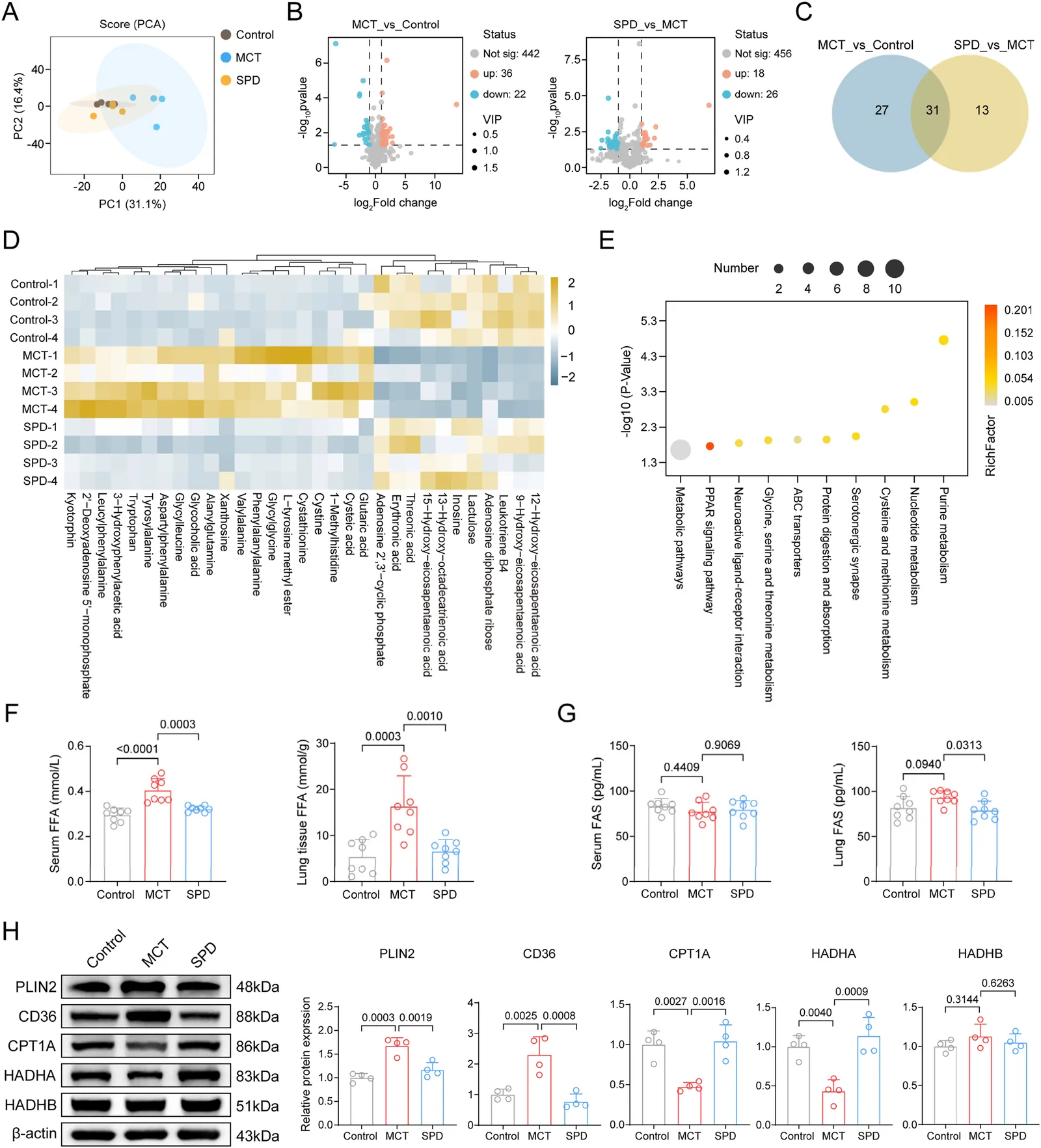
SPD attenuates lipid metabolic remodeling and fatty acid dysregulation in MCT-induced PAH rats. (A) PCA score plot of targeted metabolomics profiles from Control, MCT and SPD-treated groups (n = 4). (B) Volcano plots of differential metabolites for MCT versus Control and SPD versus MCT comparisons (VIP > 1, |log_2_FC| > 1 and *P* < 0.05; n = 4). (C) Venn diagram identifying 31 MCT-associated differential metabolites modulated by SPD. (D) Heatmap of the 31 SPD-modulated MCT-associated differential metabolites. (E) KEGG pathway enrichment analysis of these 31 metabolites. (F) Free fatty acid (FFA) levels in serum and lung tissue (n = 8). (G) Fatty acid synthase (FAS) levels in serum and lung tissue (n = 8). (H) Western blot analysis and quantification of PLIN2, CD36, CPT1A, HADHA and HADHB protein levels in lung tissue (n = 4). Data are presented as mean ± SD.

Heatmap analysis showed that these metabolites were altered in the MCT group and were partially shifted toward the Control pattern after SPD treatment (Fig. 2D). KEGG pathway analysis of these 31 metabolites identified several altered metabolic pathways, including PPAR signaling pathway, cysteine and methionine metabolism, purine metabolism, nucleotide metabolism, and other metabolism-related pathways (Fig. 2E). Among these pathways, PPAR signaling was considered particularly relevant to subsequent analysis because of its known role in fatty-acid sensing and lipid metabolic regulation. In addition, the SPD-modulated metabolite set included several fatty-acid-derived lipid mediators, including leukotriene-, prostaglandin-, and hydroxy-fatty-acid-related metabolites. These findings suggested that MCT-induced PAH may involve altered fatty-acid/lipid metabolic regulation, prompting us to further examine fatty-acid availability and synthesis-related changes in lung tissue.

We therefore measured free fatty acid (FFA) levels and fatty acid synthase (FAS) expression in serum and lung tissue homogenates. FFA levels were increased in the MCT group compared with the Control group, whereas SPD treatment reduced this increase (Fig. 2F). In contrast, FAS levels were not increased in the MCT group compared with the Control group (Fig. 2G), suggesting that the increase in FFA levels was not primarily accompanied by enhanced fatty-acid synthesis as assessed by FAS. Western blot analysis further showed that PLIN2 and CD36 were markedly upregulated in the MCT group, indicating enhanced lipid-droplet-associated storage and fatty-acid uptake. In contrast, CPT1A, a rate-limiting enzyme involved in mitochondrial fatty-acid entry for β-oxidation, and HADHA, the α-subunit of mitochondrial trifunctional protein, were downregulated, whereas HADHB levels remained largely unchanged. SPD treatment suppressed PLIN2 and CD36 expression and restored CPT1A and HADHA levels (Fig. 2H).

Together, these results indicate that MCT-induced PAH is associated with increased FFA accumulation, enhanced fatty-acid uptake/storage-related protein expression, and impaired FAO-associated protein expression in lung tissue. SPD alleviated these fatty-acid-handling and FAO-associated metabolic disturbances, providing a rationale for further mechanistic investigation in pulmonary microvascular endothelial cells.

### 3.3 SPD attenuates lipid metabolic disorder by improving FAO-related function

Metabolomic analysis using the H800 platform indicated that SPD alleviated MCT-induced lipid metabolic disorders. Fig. 3A summarizes fatty acid uptake, synthesis, storage, and FAO processes. BODIPY staining of pulmonary microartery tissues (Fig. 3B) showed abundant lipid droplets in pulmonary microvascular regions of the MCT group, which were reduced after SPD treatment. Subsequently, we employed an in vitro model by stimulating primary rat PMVECs with PA to mimic the lipotoxic environment of PAH. Immunofluorescence staining results confirmed the model's validity, as PA stimulation led to a substantial increase in the release of endothelial injury markers vWF and ET-1. Treatment with 5 μM and 10 μM SPD inhibited this excessive release (Fig. 3C). A supplementary exposure-control experiment further showed that exogenous SPD treatment increased intracellular SPD content in PMVECs within 2 h (Fig. S2), supporting the biological relevance of the in vitro treatment conditions used in this study. Consistent with this, FAO assay results (Fig. 3D) demonstrated that PA-induced lipotoxicity significantly suppressed FAO levels, which were markedly restored by 10 μM SPD treatment. Western blot analysis corroborated these findings: PA stimulation increased PLIN2 and CD36 expression and decreased CPT1A levels, changes that were significantly reversed by SPD treatment (Fig. 3E).

**Fig. 3:**
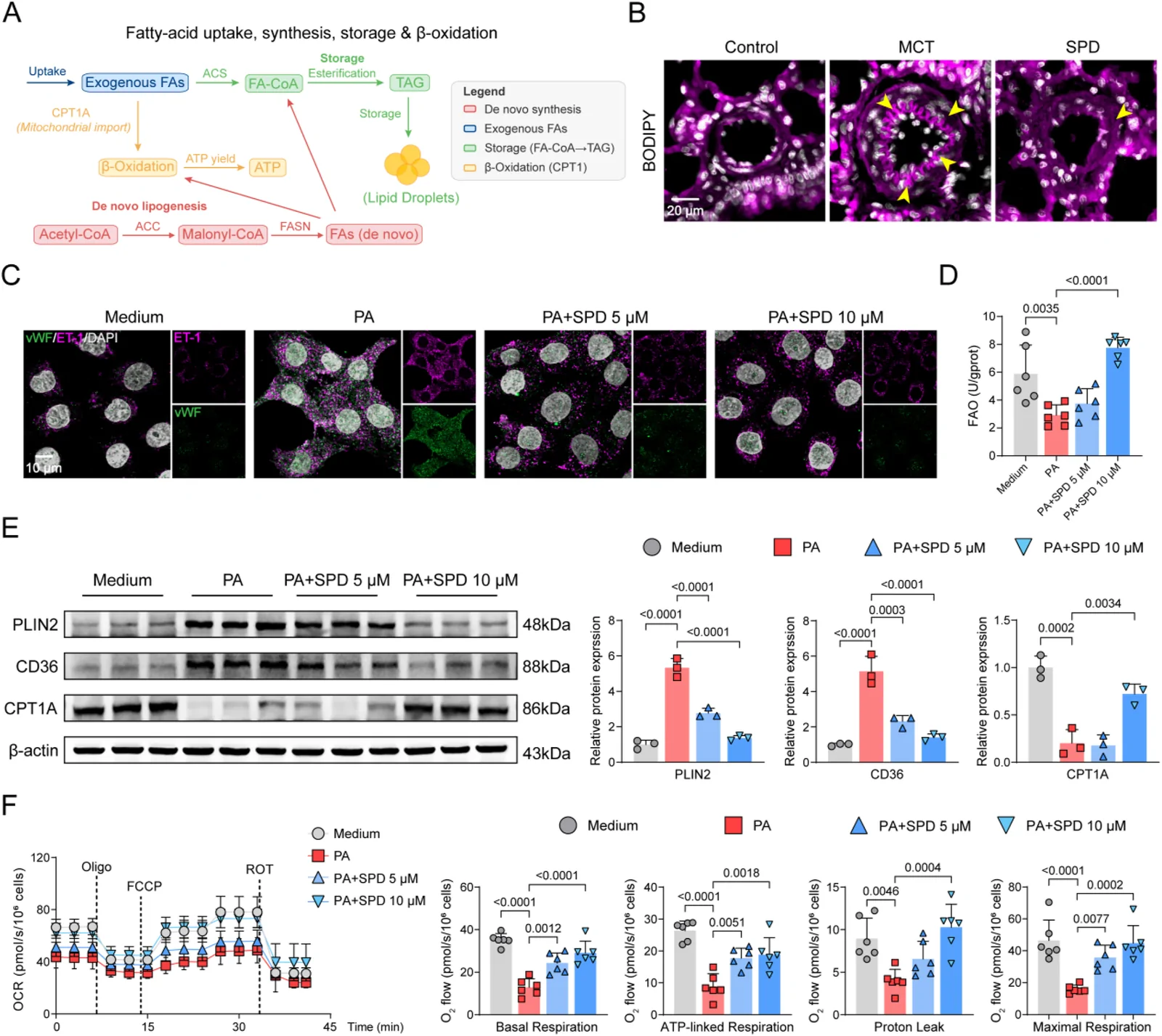
SPD improves FAO-related function and mitochondrial respiration under lipotoxic stress. (A) Schematic overview of fatty acid uptake, synthesis, storage, and beta-oxidation. (B) BODIPY 493/503 staining of lipid droplets in pulmonary microvascular regions (n = 3). (C) Immunofluorescence staining of vWF and ET-1 in PA-stimulated PMVECs treated with SPD (n = 3). (D) Colorimetric FAO activity assay in PMVECs (n = 6). (E) Protein expression levels of PLIN2, CD36, and CPT1A in PMVECs (n = 3). (F) Mitochondrial OCR parameters measured by high-resolution respirometry in PMVECs (n = 6). Data are presented as mean ± SD.

Given the central role of mitochondria in FAO and energy production, we further evaluated mitochondrial respiratory function (Fig. 3F). PA stimulation significantly reduced PMVECs' basal respiration, ATP-linked respiration, proton leak, and maximal respiratory capacity. Treatment with different concentrations of SPD (5 μM, 10 μM) restored these parameters of mitochondrial respiration. Collectively, these results indicate that SPD likely mitigates PA-induced lipotoxicity by improving FAO-related function and mitochondrial respiration.

### 3.4 SPD alleviates lipotoxicity by modulating glycerophospholipid metabolism

To further investigate lipid remodeling under endothelial lipotoxic stress, we employed the HL2400 targeted lipidomics platform to assess the regulatory effects of SPD on PA-induced lipid metabolic disturbance in PMVECs. After peak extraction, lipid annotation, and quality-control assessment, 2171 lipid species were retained in the final HL2400 targeted lipidomics matrix for downstream analysis. PCA was used as an unsupervised overview of global lipidomic distribution, whereas differential lipid species were selected using VIP > 1, |log_2_FC| > 1, and *P* < 0.05. Comparison between the PA and Medium groups revealed 652 differentially expressed lipids (321 upregulated, 331 downregulated), the majority of which belonged to the glycerophospholipid (GP) and glycerolipid (GL) classes (Fig. 4A). In contrast, the PA + SPD versus PA comparison identified 60 differentially altered lipids (10 upregulated, 50 downregulated), with GP constituting the vast majority (Fig. 4B). This suggests that SPD specifically counteracts PA-induced perturbations in GP lipids. In total, 2171 lipid molecules were identified through full-spectrum scanning. The overall lipid profile was predominantly composed of GP (60%) and GL (30%) (Fig. 4C).

**Fig. 4:**
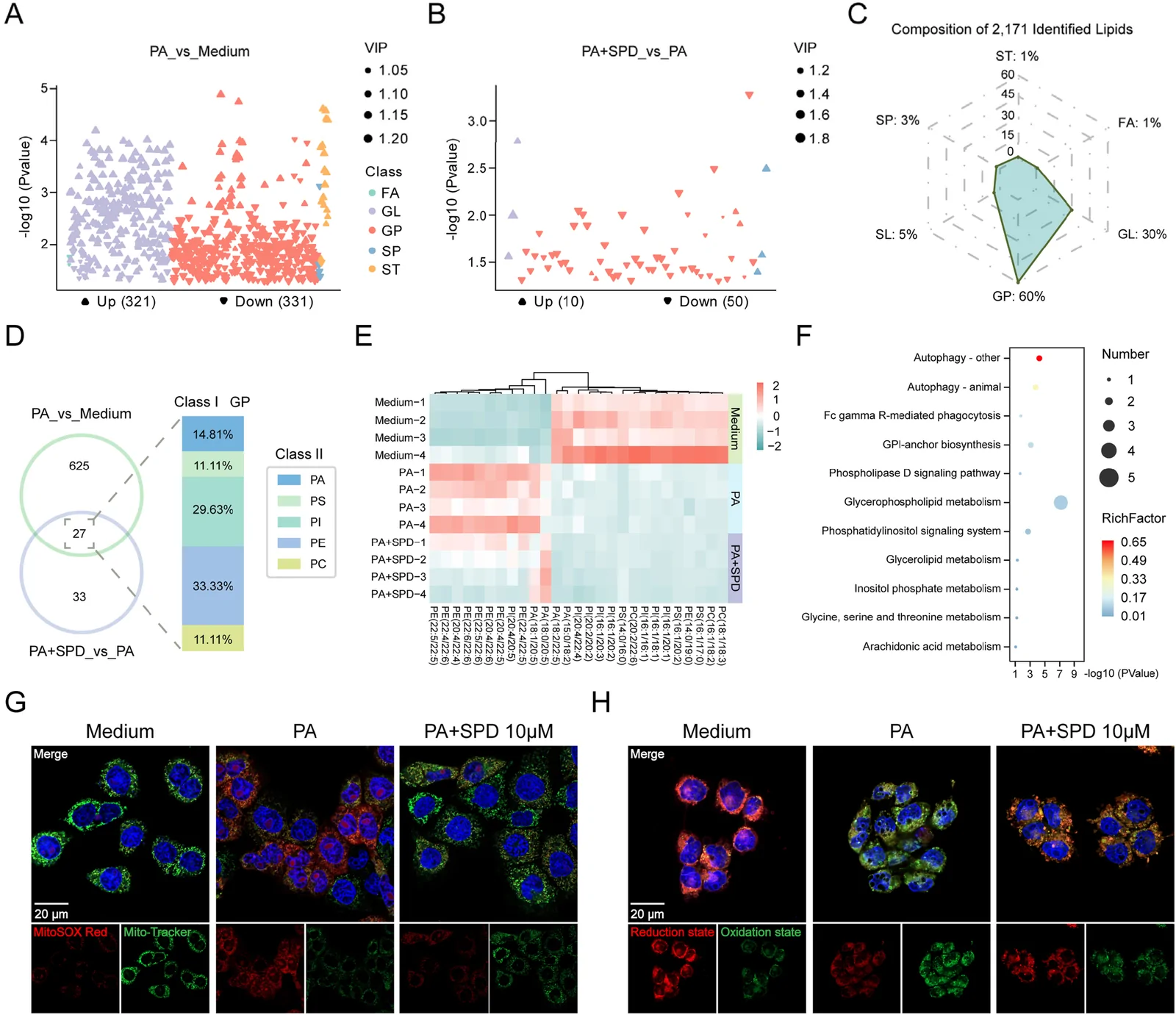
SPD-associated lipidomic remodeling is linked to reduced mitochondrial ROS and lipid peroxidation in PA-treated PMVECs. (A) Volcano plot of differential lipids between PA and Medium groups (n = 4). (B) Volcano plot of differential lipids between PA + SPD and PA groups (n = 4). (C) Lipid-class composition of all identified lipid species (n = 4). (D) Venn diagram of differential lipids and their GP subclass distribution (n = 4). (E) Hierarchical clustering heatmap of core differential lipid species (n = 4). (F) KEGG pathway enrichment analysis of core differential lipids (n = 4). (G) MitoSOX staining of mitochondrial ROS production in PMVECs (n = 3). (H) C11-BODIPY 581/591 staining of lipid peroxidation in PMVECs (n = 3).

Venn diagram analysis identified 27 common differential lipids shared between PA *vs.* Medium and PA + SPD *vs.* PA comparisons (Fig. 4D). At the primary-class level, all 27 common differential lipids belonged to GP, and subclass analysis showed that these lipids included phosphatidylethanolamine (PE), phosphatidylinositol (PI) and others. The clustering heatmap displayed the expression patterns of these 27 lipids (Fig. 4E). SPD normalized the expression pattern of PE, but did not regulate other subclasses such as PC and PI. To determine biological significance of these 27 core differential lipids, KEGG pathway enrichment analysis showed significant enrichment in critical pathways such as autophagy, FcR-mediated phagocytosis, and glycerophospholipid metabolism (Fig. 4F). These results suggest that SPD-associated correction of GP and PE-related lipid remodeling is linked to reduced PA-induced lipotoxic stress.

In parallel, direct redox assays demonstrated that SPD reduced mitochondrial ROS production (Fig. 4G) and lipid peroxidation (Fig. 4H) under lipotoxic conditions, supporting a functional link between intracellular SPD availability and redox modulation in this model. Collectively, these data identify glycerophospholipid remodeling, particularly PE-associated changes, as candidate lipid alterations linked to PA-induced lipotoxicity. Together with the subsequent redox and phenotypic analyses, these findings suggest that SPD-mediated normalization of PE-related lipid remodeling is associated with attenuation of mitochondrial oxidative stress, lipid peroxidation, and downstream lipotoxic injury.

### 3.5 SPD attenuates EndMT partly through suppression of PLIN2-associated lipotoxic signaling

Immunofluorescence analysis showed that in PA-stimulated PMVECs, PLIN2 and vimentin were highly co-expressed, whereas SPD treatment markedly reduced the expression of both PLIN2 and vimentin (Fig. 5A). In addition, GST pull-down assays confirmed a direct interaction between PLIN2 and vimentin (Fig. 5B).

**Fig. 5:**
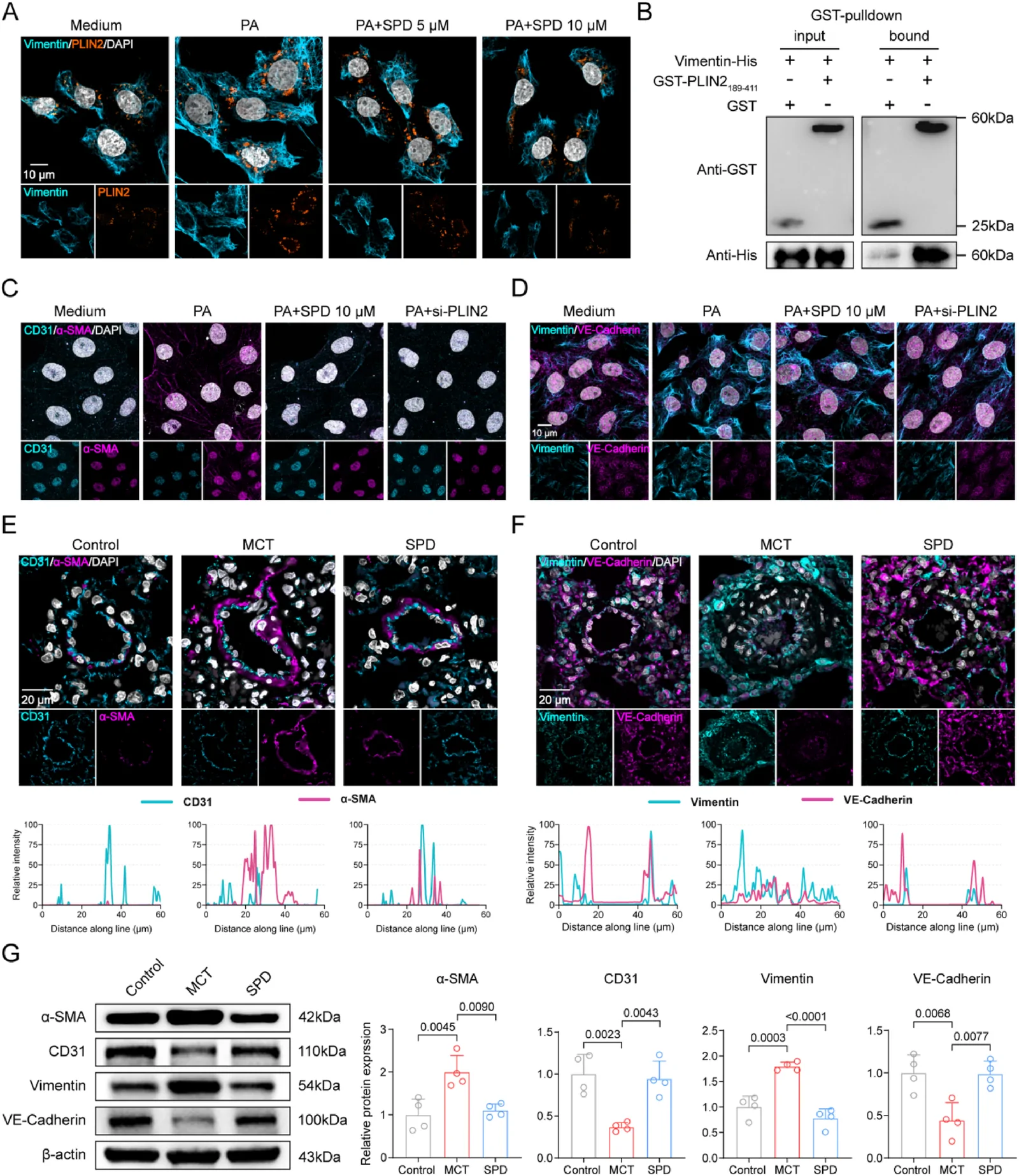
SPD suppresses PLIN2-associated lipotoxic signaling and EndMT-associated marker changes. (A) Immunofluorescence staining of PLIN2 and vimentin in PMVECs (n = 3). (B) GST pull-down assay assessing the interaction between GST-PLIN2(189-411) and His-tagged vimentin. (C, D) Immunofluorescence staining of CD31, α-SMA (C) and vimentin, VE-cadherin (D) in PA-stimulated PMVECs treated with SPD or subjected to PLIN2 knockdown (n = 3). (E, F) Immunofluorescence staining of CD31, α-SMA (E) and vimentin, VE-cadherin (F) in lung tissue (n = 3). (G) Protein expression levels of α-SMA, CD31, vimentin and VE-cadherin in lung tissue (n = 4). Data are presented as mean ± SD.

To further evaluate the effect of PLIN2 on EndMT in vitro, we performed immunofluorescence staining for EndMT markers in PMVECs from the Medium, PA, PA + SPD 10 μM, and PA + si-PLIN2 groups. As shown in Fig. 5C and D, PA stimulation decreased the endothelial markers CD31 and VE-cadherin, while increasing the mesenchymal markers α-SMA and vimentin, indicating induction of EndMT. In contrast, both SPD treatment and PLIN2 silencing partially reversed these changes, as evidenced by increased CD31 and VE-cadherin expression together with reduced α-SMA and vimentin expression. These findings suggest that both SPD and si-PLIN2 are capable of suppressing PA-induced EndMT in PMVECs.

Consistent with the in vitro findings, immunofluorescence analysis of pulmonary microarterial tissue showed similar changes in endothelial and mesenchymal markers. Compared with the control group, the MCT group exhibited a significant decrease in the endothelial markers CD31 and VE-cadherin (Fig. 5E), accompanied by a marked increase in the mesenchymal markers α-SMA and vimentin (Fig. 5F). Importantly, SPD intervention significantly attenuated these MCT-induced EndMT changes. Finally, Western blot analysis of lung tissue (Fig. 5G) demonstrated protein expression patterns of CD31, VE-cadherin, α-SMA, and vimentin that were consistent with the immunofluorescence results. Collectively, these data indicate that SPD significantly alleviates EndMT changes, at least in part through suppression of PLIN2-associated lipotoxic signaling.

### 3.6 SPD improves mitophagy-related mitochondrial quality control under lipotoxic stress

Transmission electron microscopy (TEM) analysis (Fig. 6A) revealed that cells in the Medium group exhibited no obvious abnormalities in the nucleus, chromatin, mitochondria, or endoplasmic reticulum (indicated by green arrows). In contrast, PA stimulation induced the appearance of numerous lipid droplets. In addition, some mitochondria showed marked swelling, loss of cristae structure, and dilation of the endoplasmic reticulum (indicated by red arrows). Following SPD treatment, the number of lipid droplets was reduced. Moreover, swollen mitochondria, mitochondria with disrupted cristae, and dilated endoplasmic reticulum were markedly alleviated. Concurrently, damaged mitochondria undergoing autophagic processing were observed (indicated by blue arrows).

**Fig. 6:**
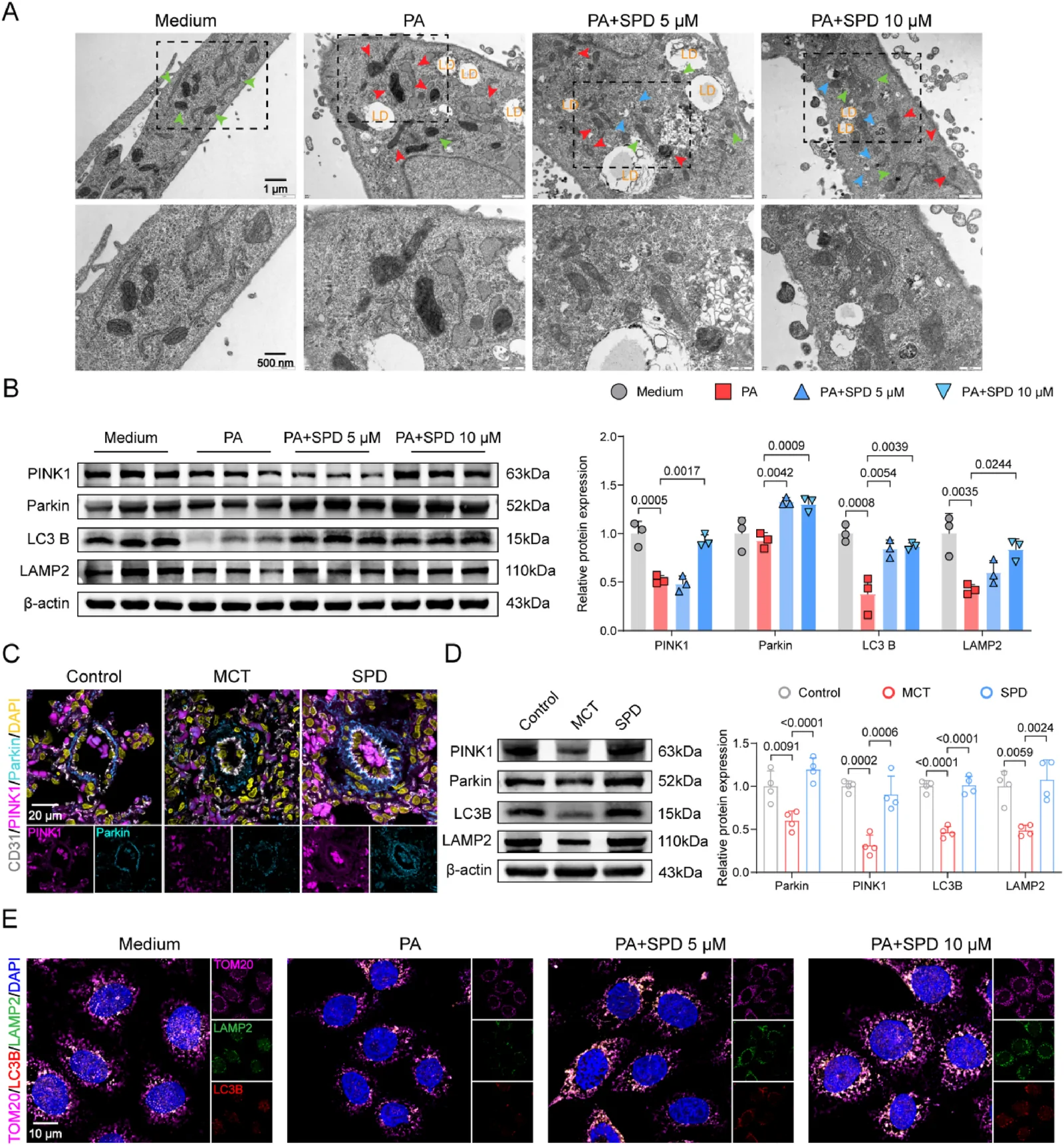
SPD improves mitophagy-related mitochondrial quality control under lipotoxic stress. (A) TEM images of PMVECs showing lipid droplets, mitochondrial morphology, and autophagic structures (n = 3). (B) Protein expression levels of PINK1, Parkin, LC3B, and LAMP2 in PMVECs (n = 3). (C) Immunofluorescence staining of CD31 together with mitophagy-related signals (PINK1 and Parkin) in pulmonary microvascular regions of lung tissue (n = 3). (D) Protein expression levels of PINK1, Parkin, LC3B, and LAMP2 in lung tissue (n = 4). (E) Immunofluorescence staining of TOM20, LC3B, and LAMP2 in PMVECs to assess mitochondrial association with autophagosome/lysosome markers (n = 3). Data are presented as mean ± SD.

We next performed Western blot analysis of mitophagy-related proteins in PMVECs (Fig. 6B). The protein expression levels of PINK1, LC3B, and LAMP2 were significantly decreased in the PA group compared with the control group, whereas SPD treatment partially increased these mitophagy-related markers. Immunofluorescence analysis of pulmonary microvascular regions in rat lung tissue (Fig. 6C) showed a similar trend. Compared with the control group, the MCT group exhibited reduced PINK1 and Parkin signals in pulmonary microvascular areas, whereas SPD treatment partially restored these signals. These findings are consistent with the involvement of the pulmonary endothelial compartment, although they do not constitute definitive endothelial-specific mechanistic validation in vivo. Consistently, Western blot analysis of lung tissue (Fig. 6D) showed that the protein expression levels of PINK1, Parkin, LC3B, and LAMP2 were significantly lower in the MCT group than in the control group, and SPD treatment increased their expression levels.

In addition, immunofluorescence staining of TOM20, LC3B, and LAMP2 in PMVECs (Fig. 6E) further supported these findings. Compared with the Medium group, PA treatment caused the TOM20-labeled mitochondrial network to become discontinuous and fragmented, suggesting impaired mitochondrial integrity with possible outer membrane damage. Meanwhile, the colocalization of LC3B and LAMP2 with TOM20-positive mitochondria was markedly reduced, consistent with impaired association of mitochondria with autophagosome/lysosome-related markers. After SPD treatment, TOM20 staining appeared more continuous, and LC3B/LAMP2 colocalization with TOM20 increased, consistent with reduced mitochondrial damage and enhanced mitophagy-related mitochondrial quality control.

Collectively, these results indicate that SPD alleviates mitochondrial lipotoxic injury and improves mitophagy-related mitochondrial quality control in PAH-related endothelial cells.

### 3.7 Biophysical validation of SPD-HADHA binding and HADHA stabilization

Molecular docking and dynamics simulations were first performed to evaluate the potential interaction between SPD and HADHA, the α-subunit of mitochondrial trifunctional protein. The predicted SPD-HADHA complex showed a stable binding conformation during the final stage of the 100-ns simulation (Fig. 7A). Residue-wise decomposition analysis identified key amino acid residues contributing to the SPD-HADHA interaction (Fig. 7B). The HADHA-SPD complex remained stable throughout the simulation, with an average RMSD of 2.96 Å for the protein backbone and 1.56 Å for SPD, indicating that SPD was retained within the predicted binding pocket without obvious drift (Fig. 7C). RMSF analysis further indicated limited local flexibility of HADHA residues in the complex (Fig. 7D). Hydrogen bond analysis showed stable interactions between SPD and HADHA, with an average donor–acceptor distance of 2.78 ± 0.09 Å and a mean bond angle of 161 ± 7.2° (Fig. 7E). MM-GBSA analysis yielded a binding free energy of −6.66 kcal/mol, supporting a favorable predicted interaction (Fig. 7F). PCA of the trajectories showed that the conformational space shifted from the initial state to a more defined cluster, suggesting formation of a stable conformational ensemble in the bound state (Fig. 7G).

**Fig. 7:**
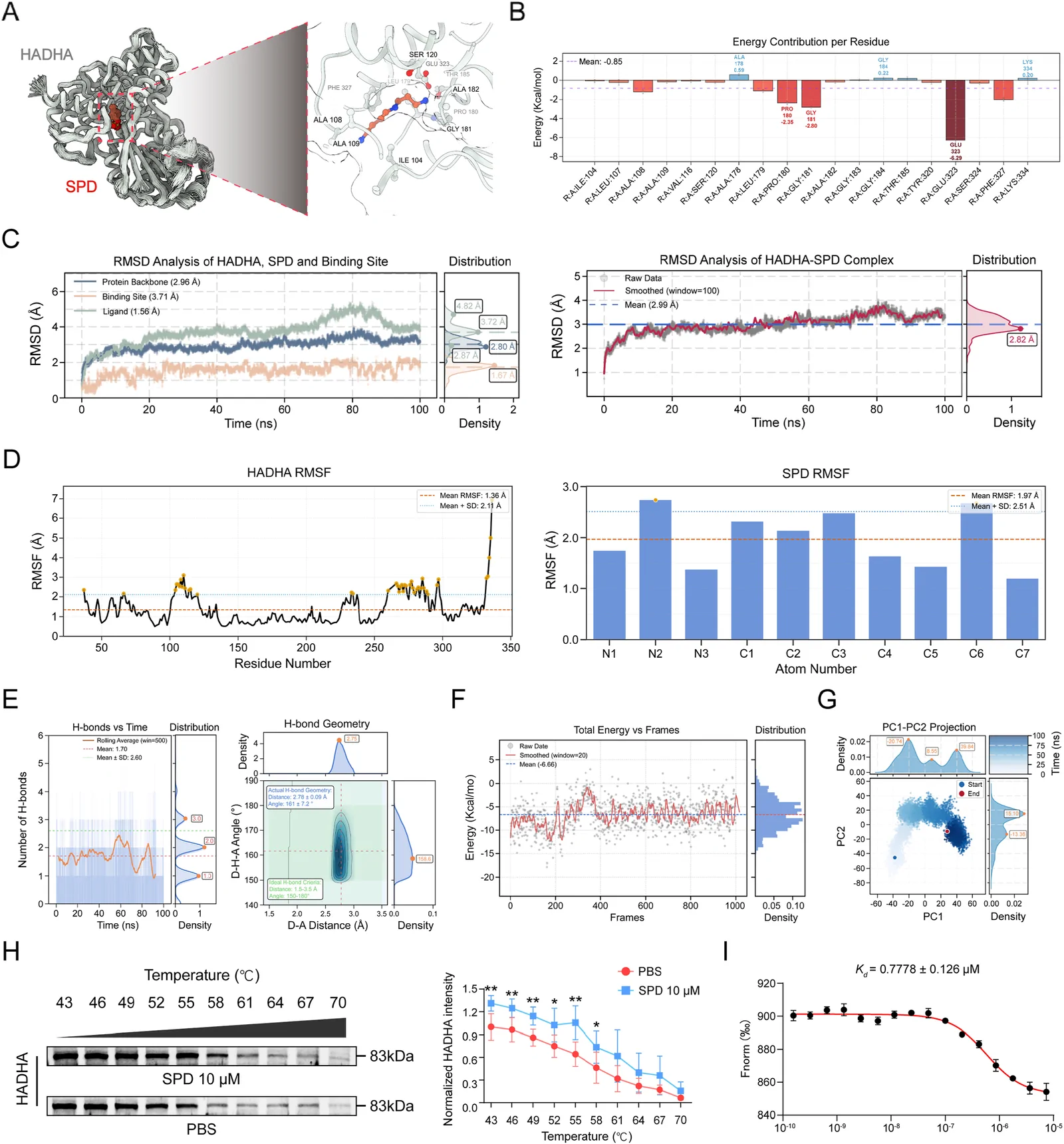
SPD-HADHA binding and HADHA stabilization. (A) Predicted structure and binding site of the HADHA-SPD complex. (B) Residue-wise decomposition of SPD-HADHA interaction energy. (C) RMSD analysis of the HADHA-SPD complex during molecular dynamics simulation. (D) RMSF analysis of HADHA residues and SPD atoms. (E) Hydrogen-bond analysis of the HADHA-SPD complex. (F) MM-GBSA binding free-energy analysis. (G) PCA of the molecular dynamics trajectory. (H) CETSA analysis showing SPD-associated HADHA thermal stabilization in PMVEC lysates (n = 6). (I) MST binding curve showing concentration-dependent interaction between SPD and recombinant human HADHA, with a calculated Kd of 0.778 μM (n = 3). Data are presented as mean ± SD.

To further validate SPD-HADHA target engagement experimentally, CETSA was performed. SPD treatment increased the thermal stability of HADHA compared with the vehicle control, supporting SPD-induced HADHA stabilization or target engagement in cell lysates (Fig. 7H). Importantly, MST analysis further demonstrated a concentration-dependent interaction between SPD and recombinant human HADHA, with a calculated Kd of 0.778 μM, providing direct biophysical evidence for SPD-HADHA binding (Fig. 7I). Together, these computational, thermal-stability, and MST data support direct physical binding between SPD and HADHA and SPD-associated stabilization of HADHA. However, direct HADHA/MTP enzymatic activity was not measured in the present study; therefore, these findings support SPD-HADHA binding and stabilization rather than direct proof of enzymatic activation.

### 3.8 HADHA knockdown attenuates the effects of SPD on FAO and redox homeostasis under lipotoxic stress

To assess the contribution of HADHA to the effects of SPD, HADHA was knocked down in PA-stimulated PMVECs, followed by evaluation of lipotoxic phenotypes, FAO-related function, mitochondrial redox injury, and related protein expression. Immunofluorescence staining showed that PA markedly increased PLIN2 and vimentin expression compared with the Medium group, whereas SPD treatment substantially reduced both signals. However, HADHA knockdown weakened the inhibitory effect of SPD on PLIN2 and vimentin (Fig. 8A).

**Fig. 8:**
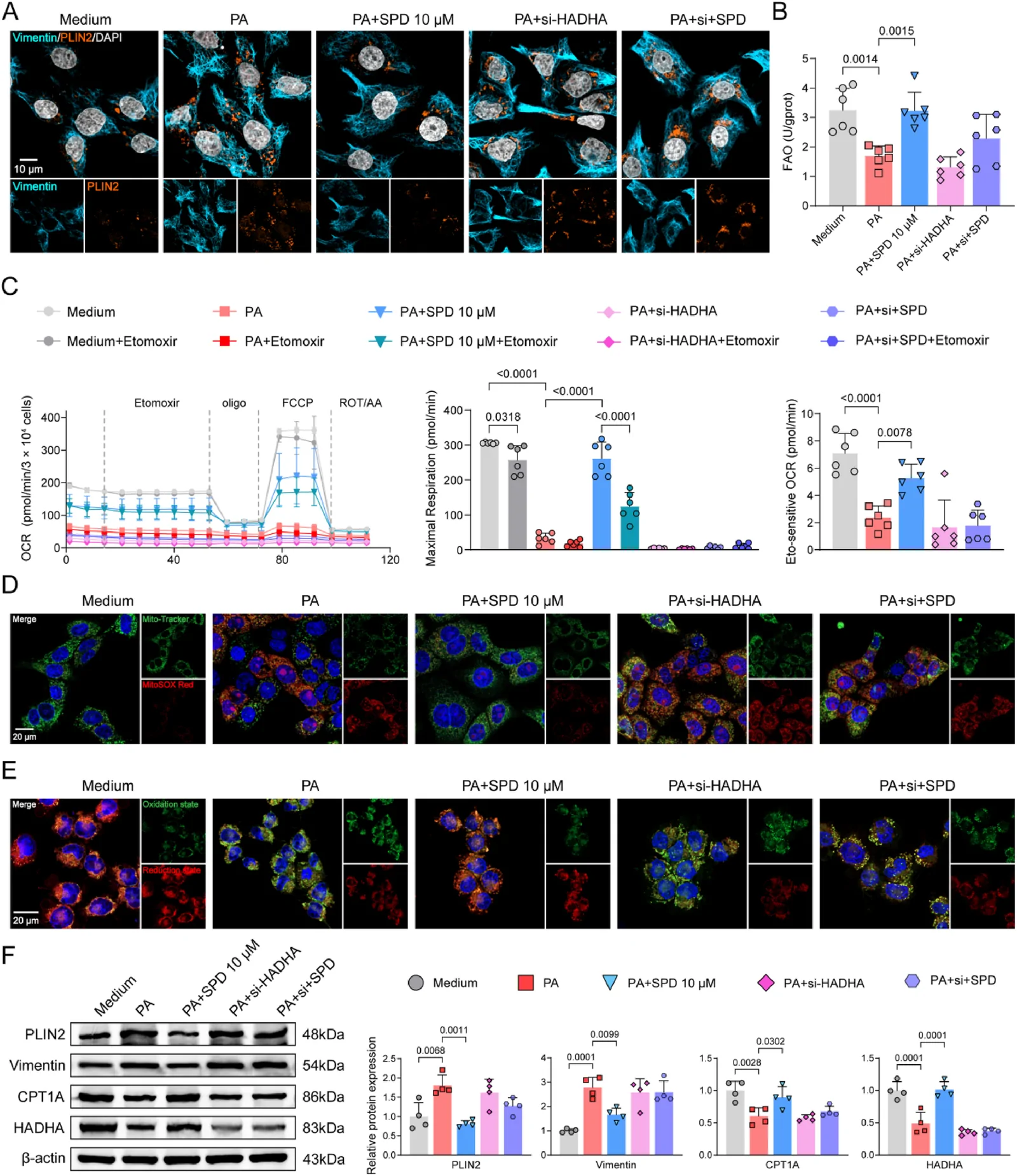
HADHA knockdown attenuates the effects of SPD on FAO-related function, mitochondrial redox homeostasis, and lipotoxic phenotypes in PMVECs. (A) Immunofluorescence staining of PLIN2 and vimentin in PA-stimulated PMVECs with or without SPD treatment and HADHA knockdown (n = 3). (B) Colorimetric FAO activity assay (n = 6). (C) Seahorse-based endogenous FAO assessment showing etomoxir-sensitive OCR as a readout of FAO-associated mitochondrial respiration (n = 6). (D) MitoSOX staining of mitochondrial ROS production (n = 3). (E) C11-BODIPY 581/591 staining of lipid peroxidation (n = 3). (F) Protein expression levels of PLIN2, vimentin, CPT1A, and HADHA (n = 4). Data are presented as mean ± SD.

Consistently, colorimetric FAO analysis showed that PA stimulation significantly reduced FAO activity, whereas SPD treatment restored FAO-related function. This restorative effect was markedly attenuated after HADHA knockdown, as FAO activity remained suppressed in the PA + si-HADHA and PA + si + SPD groups (Fig. 8B). To further evaluate FAO-associated mitochondrial respiration in live cells, we performed Seahorse-based endogenous FAO assessment. PA stimulation impaired FAO-dependent respiratory activity, whereas SPD improved etomoxir-sensitive OCR, indicating partial restoration of FAO-associated mitochondrial respiration. Importantly, HADHA knockdown markedly reduced the ability of SPD to restore FAO-dependent OCR, supporting a contribution of HADHA to this respiratory response (Fig. 8C).

We next examined how HADHA knockdown affected the redox responses to SPD. MitoSOX staining showed that PA markedly increased mitochondrial ROS production, which was reduced by SPD treatment. However, this inhibitory effect of SPD on mitochondrial ROS was substantially weakened after HADHA knockdown (Fig. 8D). Similarly, C11-BODIPY 581/591 staining showed that PA-induced lipid peroxidation was attenuated by SPD, whereas HADHA knockdown diminished this protective effect (Fig. 8E).

Western blot analysis further supported these observations. PA increased PLIN2 and vimentin expression and reduced CPT1A and HADHA expression, whereas SPD reversed these changes. In contrast, HADHA knockdown blunted the regulatory effects of SPD on these FAO- and lipotoxicity-related proteins (Fig. 8F). Collectively, these data show that HADHA knockdown markedly attenuated the effects of SPD on FAO-related function, mitochondrial ROS, lipid peroxidation, and downstream lipotoxic phenotypes in PMVECs.

## 4 Discussion

This study supports a role for HADHA in the effects of SPD on fatty acid oxidation and mitochondrial redox homeostasis in MCT-induced PAH rats and PA-stimulated PMVECs. In MCT-induced PAH rats, SPD reduced pulmonary arterial pressure, right ventricular remodeling, inflammatory activation, endothelial injury markers, and vascular remodeling. In PA-stimulated PMVECs, SPD reduced lipid accumulation, improved FAO-related mitochondrial respiration, and attenuated mitochondrial ROS production and lipid peroxidation. The relevance of HADHA to these responses is supported by MST and CETSA evidence for SPD binding and HADHA stabilization, together with the attenuation of several SPD responses after HADHA knockdown. Together, these observations link HADHA to the changes in FAO and mitochondrial redox status observed with SPD treatment.

IL-6, vWF, and ET-1 are well-established mediators involved in PAH progression and are closely linked to endothelial dysfunction and vascular remodeling [2,14,15]. Beyond their classical inflammatory or vasoactive roles, increasing evidence suggests that alterations in these factors are accompanied by broader metabolic reprogramming. IL-6 has been reported to promote lipolysis and increase free fatty acid supply, vWF is associated with dysregulated lipid handling, and ET-1 has been linked to oxidative stress–related metabolic disturbances [[16], [17], [18]]. Consistent with these observations, metabolomic profiling of lung tissues from PAH rats revealed pronounced lipid metabolic abnormalities. Excessive lipid accumulation was associated with endothelial injury and impaired FAO, findings that are in line with clinical reports describing lipid deposition and reduced FAO capacity in PAH patients [11,19]. In vitro, PA-stimulated PMVECs released large amounts of vWF, ET-1 and showed reduced FAO activity. Reduced FAO was accompanied by mitochondrial respiratory dysfunction in endothelial cells, a pattern consistent with metabolic stress in PAH.

Lipidomic analysis identified GP remodeling, particularly PE-associated changes, as a prominent feature of PA-induced endothelial lipotoxicity. PE is a major membrane phospholipid in cellular and mitochondrial membranes and is required for LC3 lipidation, linking PE metabolism to autophagy-related processes [20]. Under lipotoxic and oxidative conditions, disturbed PE homeostasis and phospholipid peroxidation may compromise membrane integrity, mitochondrial function, and mitochondrial quality-control pathways [21]. In the present study, SPD-associated correction of PE-related lipidomic changes was accompanied by reduced mitochondrial ROS, reduced lipid peroxidation, and attenuation of lipotoxic phenotypes. These findings support PE-associated remodeling as a mechanistically relevant lipid signature in this model. However, oxidized PE species were not directly quantified, and the current data do not establish PE remodeling as a causal mediator.

PLIN2 is widely recognized as a marker of intracellular lipid droplet accumulation [22], whereas vimentin is a hallmark of EndMT [23]. Previous studies have shown that lipid droplets, PLIN2, and vimentin can form functional complexes in adipocytes [24]. Based on these observations, we speculated that PLIN2 may promote lipid accumulation and facilitate EndMT during PAH progression. Indeed, immunofluorescence analysis demonstrated co-localization of PLIN2 and vimentin in PA-stimulated PMVECs, and GST pull-down assays confirmed a direct interaction between these proteins. These results suggest that PLIN2–vimentin complexes may contribute to endothelial phenotypic switching and vascular remodeling. Importantly, SPD treatment downregulated PLIN2 expression and effectively suppressed EndMT. To our knowledge, this study provides the first evidence that inhibition of PLIN2 not only alleviates lipid accumulation but also reverses EndMT-associated phenotypic changes, thereby restoring endothelial integrity in PAH.

Disordered lipid metabolism is also known to induce mitochondrial damage and impair mitophagy [[25], [26], [27]]. In the present study, mitochondrial structural abnormalities were evident in PMVECs following PA stimulation, whereas SPD treatment restored mitochondrial architecture and enhanced mitophagic activity. In rat lung tissue, immunofluorescence findings in pulmonary microvascular regions were consistent with partial restoration of mitophagy-related signaling after SPD administration. Together with the PMVEC-based data, these results support endothelial relevance of this pathway in PAH, but they do not yet establish a definitive endothelial-specific mechanism in vivo. Notably, some studies have suggested that mitochondrial autophagy in endothelial cells is activated during the process of PAH [28], while others have found that promoting mitochondrial autophagy in endothelial cells can inhibit vascular remodeling [29]. These observations raise the possibility that mitophagy is dynamically regulated during disease progression. We speculate that, during the early compensatory phase of PAH, mitophagy is upregulated to facilitate removal of damaged mitochondria, whereas prolonged metabolic and oxidative stress in later stages may exhaust the autophagic machinery, resulting in mitochondrial redox imbalance. In this context, PINK1/Parkin signaling is generally recognized as a mitochondrial quality-control pathway activated in response to damaged or depolarized mitochondria [[30], [31], [32]]. Thus, the SPD-associated increase in PINK1/Parkin signals, together with enhanced TOM20/LC3B/LAMP2 colocalization, improved mitochondrial morphology, and reduced mitochondrial redox injury, supports improved mitophagy-related mitochondrial quality control under lipotoxic stress. Consistently, HADHA has been implicated in long-chain fatty acid-induced autophagy and has been identified as a Parkin-interacting protein in independent studies, supporting a plausible functional link between fatty acid oxidation and mitochondrial quality control [33,34].

MTP is associated with the inner mitochondrial membrane and catalyzes three reactions in the long-chain FAO cycle. MTP is a hetero-octamer consisting of four α subunits (HADHA) and four β subunits (HADHB). HADHA catalyzes the long-chain 2,3-enoyl-CoA hydratase (ECH) and long-chain 3-hydroxyacyl-CoA dehydrogenase (LCHAD) reactions, whereas HADHB catalyzes the 3-ketoacyl-CoA thiolase (KACT) reaction [33]. A previous study reported that SPD acutely increases FAO and ATP production in CD8^+^ T cells by directly activating MTP [13]. In addition, SPD supplementation has been linked to protection against thoracic aortic dissection, possibly through restoration of HADHB-dependent FAO [35]. In the present study, changes in HADHA and fatty acid oxidation in pulmonary endothelial cells were accompanied by altered mitochondrial redox homeostasis, lipid peroxidation, EndMT markers, and markers of mitophagy in the PAH setting.

In the present PAH model, HADHA and HADHB were differentially affected: HADHA was markedly reduced, whereas HADHB was not significantly changed. This observation led us to focus on HADHA as a disease-relevant MTP subunit in pulmonary endothelial lipotoxicity. Molecular dynamics simulations predicted a stable interaction between SPD and HADHA, and CETSA showed increased thermal stability of HADHA in the presence of SPD, supporting SPD-associated target engagement or protein stabilization. Importantly, MST provided direct biophysical evidence for SPD-HADHA binding, with a calculated Kd of 0.778 μM. Together with the HADHA knockdown data, these findings support direct binding between SPD and HADHA and indicate that HADHA contributes to the effects of SPD on FAO and redox homeostasis under endothelial lipotoxic stress.

Nevertheless, MST demonstrates physical binding rather than enzymatic activation. Therefore, although our data support SPD-HADHA binding and a contribution of HADHA to the functional effects of SPD, direct evidence that SPD enhances HADHA/MTP catalytic activity in the PAH endothelial context remains to be obtained. Future studies using purified MTP/HADHA enzymatic assays will be required to determine whether SPD binding directly enhances HADHA catalytic function. Collectively, our data support direct binding and stabilization of HADHA by SPD, while SPD treatment was accompanied by improved FAO-related function, reduced lipotoxicity, and enhanced mitophagy-related mitochondrial quality control (Fig. 9).

**Fig. 9:**
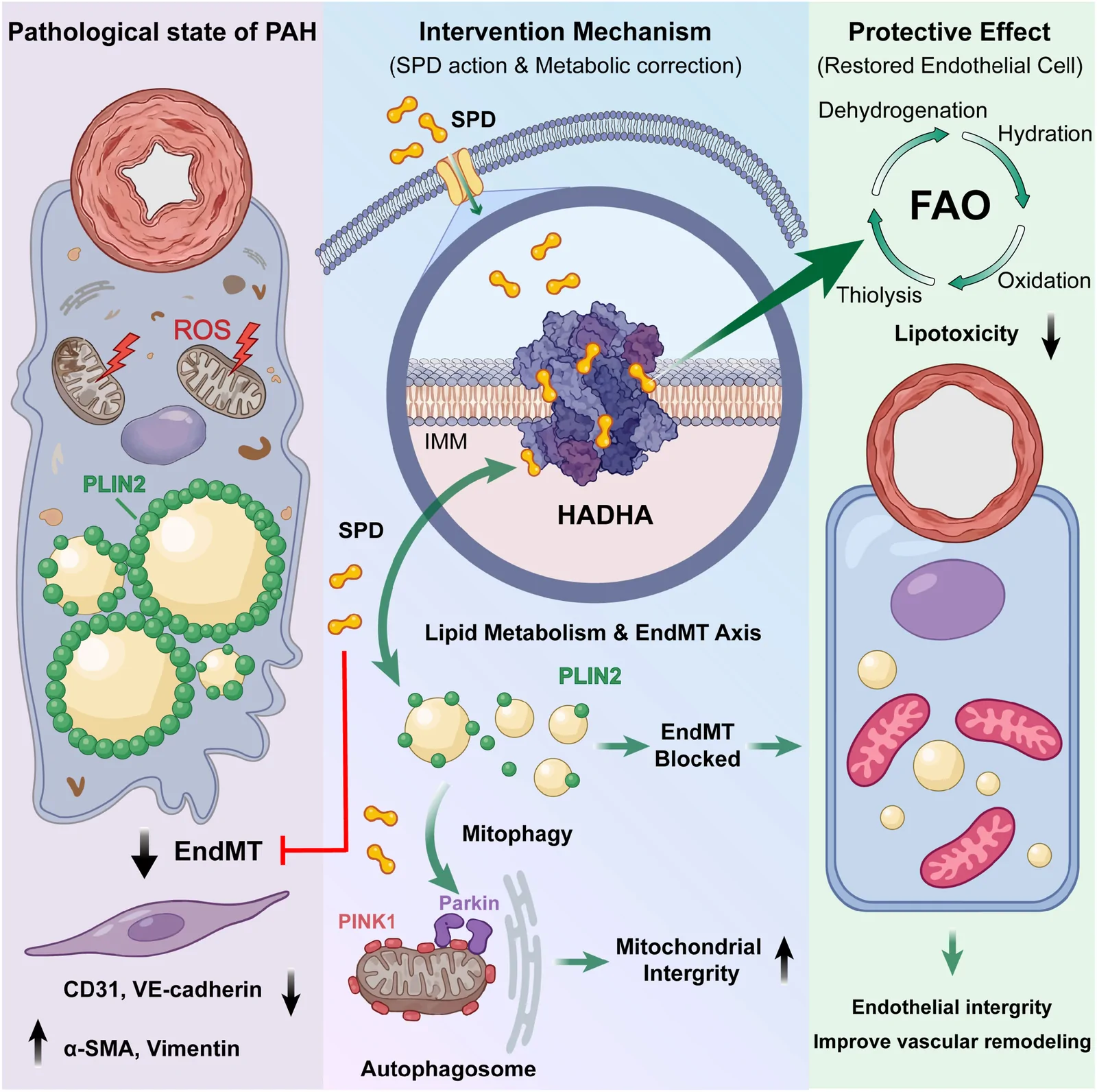
Proposed model of endothelial protection by SPD in MCT-induced PAH and PA-related lipotoxic injury. SPD binds to and stabilizes HADHA, while SPD treatment is accompanied by improved fatty acid oxidation and mitochondrial redox homeostasis, attenuation of EndMT-associated changes, and improved mitophagy-related mitochondrial quality control.

Several limitations should be acknowledged. First, although intracellular SPD levels increased under our in vitro treatment conditions (Fig. S2), the present study did not establish a quantitative pharmacokinetic–pharmacodynamic relationship, and circulating SPD exposure after intraperitoneal administration was not determined. Second, MST provided direct biophysical evidence for SPD-HADHA binding, but direct HADHA/MTP enzymatic activity was not measured; therefore, whether SPD binding directly enhances HADHA catalytic function in the PAH endothelial context remains to be clarified. Third, although PE-associated lipid remodeling was linked to redox injury and lipotoxic phenotypes, oxidized PE species were not directly quantified. Finally, while PMVEC-based experiments support endothelial relevance, the current in vivo tissue-level analyses do not establish definitive endothelial-specific causality. Future studies using endothelial-specific approaches will be required to further define the cell-type-specific contribution of HADHA to the effects of SPD.

## 5 Conclusion

In conclusion, SPD attenuated endothelial injury and pulmonary vascular remodeling in MCT-induced PAH. SPD also improved FAO and mitochondrial redox homeostasis. In parallel, direct binding to and stabilization of HADHA were supported by MST and CETSA, respectively. HADHA knockdown attenuated several metabolic and redox responses to SPD in PMVECs. Together, these findings support a role for HADHA in the endothelial effects of SPD in experimental PAH.

## CRediT authorship contribution statement

**Xinyu Bai:** Writing – review & editing, Writing – original draft, Visualization, Software, Funding acquisition, Data curation, Conceptualization. **Keke Tian:** Writing – original draft, Visualization, Data curation. **Shiyuan Song:** Validation, Data curation. **Xiuming Rao:** Validation, Data curation. **Gang Xu:** Validation, Data curation. **Erlong Zhang:** Visualization, Software. **Bao Liu:** Supervision. **Yan Wang:** Supervision. **Yuqi Gao:** Writing – review & editing, Resources, Project administration, Funding acquisition, Conceptualization.

## Ethics declaration

This study was conducted in accordance with the ARRIVE (Animal Research: Reporting of In Vivo Experiments) guidelines. This study was approved by the Institutional Animal Care and Use Committee of Yu-Yue Pathology Scientific Research Center. (Approval No. CARE-YYLAB-D-2026-001)

## Declarations

All authors have approved the manuscript and agree with its submission to Redox Biology. The study was conducted in accordance with the relevant ethical guidelines, and all required ethical approvals are reported in the manuscript.

## Declaration of generative AI use

Generative AI tools were used solely for grammar correction. No content generation, data analysis, or figure creation involved AI assistance.

## Funding

This work was supported by the 10.13039/501100001809National Natural Science Foundation of China (grant number 81830062), Innovative Drug Research and Development — National Science and Technology Major Project (grant number 2025ZD1802901), and Natural Science Foundation of Chongqing Science and Technology Bureau (grant number CSTB2024NSCQ-MSX1047).

## Declaration of competing interest

The authors declare that they have no known competing financial interests or personal relationships that could have appeared to influence the work reported in this paper.

## Data Availability

H800-targeted metabolomics data are available via MetaboLights with the identifier MTBLS13809. Lipidomics data for HL2400 are available via MetaboLights with the identifier MTBLS13808. Source data are provided with this paper. The data that support the findings of this study are available from the corresponding author upon reasonable request.
