# Supplementary material for: Spermidine attenuates monocrotaline-induced pulmonary arterial hypertension with HADHA-associated improvements in fatty acid oxidation and mitochondrial redox homeostasis
Source: Redox Biol. 2026 Sep 17;97:104392. doi: 10.1016/j.redox.2026.104392 (PMC13626935; doi:10.1016/j.redox.2026.104392)
Supplement: Supplementary file 1 [file mmc1.docx]

**Supplementary Information**

**Supplementary Methods**

**Supplementary Method S1. Additional in vivo baseline-control experiment with SPD alone**

To determine whether SPD administration alone affects baseline physiological and hemodynamic parameters in healthy rats, an additional supplementary in vivo cohort was established. Male Sprague-Dawley rats were assigned to a Control group and a Control+SPD group. Rats in the Control+SPD group received intraperitoneal SPD administration (5 mg/kg/day) for 28 days, consistent with the dosing regimen used in the main in vivo study, whereas rats in the Control group received the corresponding vehicle under the same schedule. No MCT challenge was administered in this supplementary cohort.

At the experimental endpoint, baseline hemodynamic and physiological parameters were assessed using the same procedures described in the main Methods, including right ventricular pressure recording, RVSP and mPAP quantification, and Fulton’s index analysis. The resulting data are presented in Fig. S1.

**Supplementary Method S2. Measurement of intracellular spermidine content in PMVECs after short-term exogenous SPD treatment**

PMVECs were cultured as described in the main Methods and treated with exogenous spermidine for 2 h at the concentrations indicated in Fig. S2. Cells were then rapidly washed with ice-cold PBS to remove extracellular SPD and harvested for intracellular SPD measurement. SPD stock solution (1 mg/mL) was prepared in methanol. Tolbutamide (internal standard, IS) stock solution (1 mg/mL) was prepared in DMSO, and the IS working solution (750 ng/mL) was prepared in 0.5% formic acid in methanol. Calibration standards (100–5000 ng/mL; 100, 200, 400, 800, 1600, 2500, 4000, and 5000 ng/mL) were prepared by serial dilution in 80% methanol-water. QC samples were prepared at 100 ng/mL (LLOQ), 300 ng/mL (LQC), 1500 ng/mL (MQC), and 3750 ng/mL (HQC).

**Supplementary Results**

**Supplementary Result S1. SPD alone does not significantly affect baseline hemodynamic parameters in healthy rats**

To further determine whether the protective effects observed in the main study could be attributed to nonspecific baseline hemodynamic alterations caused by SPD itself, an additional supplementary control experiment was performed in healthy rats receiving SPD alone. As shown in Fig. S1A, continuous right ventricular pressure recordings from the Control+SPD group exhibited stable waveforms comparable to those of the untreated Control group. Quantitative analysis further demonstrated that SPD administration alone did not significantly alter RVSP or mPAP (Fig. S1B). Likewise, Fulton’s index showed no detectable difference between the Control and Control+SPD groups (Fig. S1C). These findings support that, under the present experimental conditions, SPD administration alone does not significantly disturb baseline physiological or hemodynamic status in healthy rats, thereby supporting the interpretation that the beneficial effects observed in the MCT+SPD group primarily reflect disease-modifying actions.

**Fig. S1. Additional in vivo baseline-control experiment with SPD alone.** (A) Representative right ventricular pressure traces from the Control and Control+SPD groups. (B) Quantification of RVSP and mPAP. (C) Fulton’s index analysis. SPD was administered intraperitoneally at 5 mg/kg/day for 28 days, consistent with the dosing regimen used in the main in vivo study. Data are presented as mean ± SD (n = 6).

**Supplementary Result S2. Exogenous SPD treatment increases intracellular SPD content in PMVECs**

To support interpretation of cellular SPD exposure in vitro, intracellular SPD levels were measured in PMVECs after 2 h of exogenous treatment. As shown in Fig. S2, compared with the Medium group, treatment with SPD 10 μM significantly increased intracellular SPD content. Under lipotoxic conditions, the PA group showed lower intracellular SPD levels, whereas both PA + SPD 5 μM and PA + SPD 10 μM significantly increased intracellular SPD content relative to the PA group. These findings confirm that exogenous SPD treatment effectively elevates intracellular SPD availability in PMVECs under the conditions used in the main study.

**Fig. S2. Intracellular SPD content in PMVECs after 2 h exogenous SPD treatment.** Data are presented as mean ± SD (n = 6).

**Supplementary Result S3. HADHA knockdown attenuates the effect of SPD on basal mitochondrial respiration in PMVECs**

To further support the Seahorse-based FAO assessment shown in the main figures, basal mitochondrial respiration was analyzed as an additional respiratory parameter. As shown in Fig. S3, PA stimulation markedly reduced basal respiration in PMVECs, indicating impaired mitochondrial respiratory function under lipotoxic stress. SPD treatment partially restored basal respiration. However, HADHA knockdown weakened this restorative effect, as basal respiration remained suppressed in the PA+si-HADHA and PA+si-HADHA+SPD groups. These findings further indicate that HADHA knockdown attenuates the effect of SPD on mitochondrial respiratory function.

**Fig. S3. Basal respiration from Seahorse-based endogenous FAO assessment in PMVECs.** Data are presented as mean ± SD (n = 6).

**Supplementary Table**

**Supplementary Table S1. Antibody information used in this study**

| **Antibody** | **Company** | **Catalog** | **WB Dilution** | **IF/IHC Dilution** |
| --- | --- | --- | --- | --- |
| β-actin | Affinity | T0022 | 1:10000 | N/A |
| CD36 | Affinity | DF13262 | 1:1000 | N/A |
| CD31/PECAM-1 | Bioworld Tech | BS1574 | 1:500 | 1:100 |
| Perilipin-2 | Proteintech | 15294-1-AP | 1:4000 | 1:400 |
| CPT1A | Proteintech | 15184-1-AP | 1:8000 | N/A |
| HADHA | Proteintech | 10758-1-AP | 1:6000 | N/A |
| HADHB | Servicebio | GB114025 | 1:6000 | N/A |
| Vimentin | Affinity | BF8006 | 1:2000 | 1:300 |
| VE-cadherin | Proteintech | AF6265 | 1:5000 | 1:250 |
| PARK2/Parkin | Proteintech | 14060-1-AP | 1:4000 | 1:250 |
| Pink1 | Proteintech | 23274-1-AP | 1:2000 | 1:1000 |
| LC3B | Affinity | AF4650 | 1:1000 | N/A |
| LAMP2 | Immunoway | YT5711 | 1:1000 | N/A |
| α-SMA | BOSTER | BM0002 | 1:1000 | 1:500 |
| Von Willebrand Factor | Servicebio | GB11020 | N/A | 1:500 |
| Endothelin 1 | Invitrogen | MA3-005 | N/A | 1:250 |
| Goat Anti-Mouse IgG (H+L) HRP | Affinity | S0002 | 1:20000 | N/A |
| Goat Anti-Rabbit IgG (H+L) HRP | Affinity | S0001 | 1:10000 | N/A |
| Alexa Fluor 488 | Invitrogen | A11001 | N/A | 1 µg/mL |
| Alexa Fluor 647 | Invitrogen | A21244 | N/A | 4 µg/mL |

**Supplementary Table S2. Summary of targeted metabolomics and lipidomics workflows**

| **Item** | **H800 targeted metabolomics** | **HL2400 targeted lipidomics** |
| --- | --- | --- |
| Sample type | Rat lung tissue | PMVECs |
| Company/platform | H800 platform, Shanghai Applied Protein Technology | HL2400 platform, Shanghai Applied Protein Technology |
| Platform/library coverage | 765 measurable metabolites | 2,464 candidate lipid entries |
| Final analysis matrix | 500 measured metabolites | 2,171 lipid species |
| Category/class annotation | Superclass, class, subclass, and direct-parent annotations | Six major lipid categories and 25 lipid classes |
| Extraction method | Methanol/acetonitrile/water extraction solvent (2:2:1, v/v/v) | Methyl tert-butyl ether extraction |
| Internal standards | Stable-isotope-labeled internal standards | Internal standards |
| LC system | Agilent 1290 Infinity LC UHPLC | Shimadzu LC-30AD UHPLC |
| MS platform | 6500+ QTRAP mass spectrometer (AB SCIEX) | 6500+ QTRAP mass spectrometer (AB SCIEX) |
| Chromatographic separation | HILIC and C18 columns | C18 and Amino columns |
| Ionization/acquisition | Positive/negative ESI-MRM | Positive/negative ESI-MRM |
| Data processing software | MultiQuant or Analyst | Sciex OS |
| Quantification basis | Peak extraction, peak-area/internal-standard ratios, and calibration curves | Chromatographic peak integration and internal standards |
| Annotation basis | Retention time, MRM transitions, standard compounds, KEGG ID, HMDB ID, superclass, class, subclass, and direct parent | Retention time, MRM transitions, targeted lipid library, lipid class annotation, and internal standards |
| QC design | Pooled QC samples inserted into the analytical sequence | Pooled QC samples inserted into the analytical sequence |
| QC evaluation | TIC overlap, QC PCA clustering, Pearson correlation, Hotelling’s T², multivariate control charts, and QC RSD/CV assessment | TIC overlap, QC PCA clustering, Pearson correlation, Hotelling’s T², multivariate control charts, and QC RSD/CV assessment |
| Unsupervised overview | PCA | PCA |
| VIP source | OPLS-DA | OPLS-DA |
| Differential criteria | VIP > 1, \|log_2_FC\| > 1, and *P* < 0.05 | VIP > 1, \|log_2_FC\| > 1, and *P* < 0.05 |
